# A scoping review of the prevalence of musicians’ hearing loss

**DOI:** 10.3389/fpubh.2025.1472134

**Published:** 2025-02-13

**Authors:** Carl Firle, Antonia Helen Richter

**Affiliations:** ^1^Federal Institute for Occupational Safety and Health, Berlin, Germany; ^2^Verband deutscher Betriebs- und Werksärzte e.V., AG Bühnen und Orchester, Magdeburg, Germany

**Keywords:** noise-induced hearing loss, music-induced hearing loss, musician, orchestra, hearing protection

## Abstract

**Introduction:**

Hearing loss is the most commonly recognized occupational disease in Germany. Musicians are also affected, as playing classical music can expose them to high or very high sound volumes. With this scoping review, we aimed to assess the prevalence of noise-induced hearing loss among professional musicians and evaluate its characteristics.

**Methods:**

The databases such as MEDLINE, Embase, Cochrane Library, and Google Scholar were searched using the terms *(hearing loss OR hearing impairment OR hearing difficulties OR acoustic trauma) AND (musician)* on 14 August 2023 and 2 January 2025. Only original studies with audiometric examination results were included.

**Results:**

A total of 79 studies were retrieved for descriptive analysis. The median number of participants was 52 (IQR 30-109). The majority of the retrieved studies included participants with at least 5 years of experience as practicing musicians. The proportion of men was significantly higher than that of women, with the median_men portion_ of 69% (IQR 53–83%). Students were a common study population, indicating that the data on older and retired musicians were either rare or missing. As a result, the lifetime prevalence of hearing loss in musicians could not be determined. The data analysis showed an increased risk of hearing loss >15–20 dB in the frequency range of 4,000–6,000 Hz among participants in the classical genre group. Studies with participants having normal hearing were also found within that genre. Rock, pop, and jazz musicians had an increased risk of hearing loss >20 dB in the frequency range of 3,000–8,000 Hz. The data for military and marching band music and traditional music genres were limited. The retrieved studies indicated a higher risk of hearing loss >20 dB in the frequency range of 4,000–6,000 Hz. A total of 17 studies adjusted the audiogram results for age, 2 did not, and 59 had no report. Data extraction yielded a prevalence of notch configurations in 20–50% of the classical musicians, with hearing loss affecting 5–70% of them. Up to 40% of rock, pop, and jazz musicians showed notch configurations, with 20–60% experiencing hearing loss.

**Conclusion:**

Overall, a definitive assessment of the prevalence of musicians’ hearing loss cannot be drawn from the available data. Prospective, longitudinal studies with reliable sample sizes and representative populations are essential. A multicenter study would also be valuable.

## Introduction

Noise-induced hearing loss is a widespread condition in occupational health (1). The WHO attributes 16% of global hearing loss to occupational noise exposure (2). In Germany, noise-induced hearing loss is the most commonly recognized occupational disease (3) and is often a result of chronic occupational noise exposure and insufficient protection measures (4). In addition, musicians can be affected, even though music is a very special type of noise (5). Occupational examinations reveal an elevated risk of hearing loss for musicians playing in an orchestra: 8 h exposure levels may exceed L_Aeq8h_ 85 dB(A) or peak values of 137 dB(C), depending on the instrument and orchestra position (6). The law mandates that at these noise levels, employers must provide protective measures (7, 8), which are often inappropriate for musical activities. For instance, hearing protection devices can distort the sound frequency range (9) and lead to the occlusion effect, which affects musicians’ perception of the music (10). Thus, the use of hearing protection devices is low among musicians (11). Partition and sound baffles may worsen sound exposure through reflection effects or by causing inhomogeneous sound dispersion (12). Substituting loud sections of music is not an option, and administrative measures to reduce the volume during rehearsal or concerts are limited.

Given these facts, it is important to determine whether musicians have an increased prevalence of music-induced hearing loss. Previous studies have provided an overview of the topic but lack a systematic literature search. For instance, Marquard and Schäcke reported in detail in 1998 on the heterogeneity of the published studies assessing musicians’ hearing loss (13). They found major differences in outcome parameters, methodical approaches, examination conditions, and participant groups. More recent reviews assessed a broad range of musicians’ diseases and briefly reported on hearing loss (14, 15). Other reviews focused on noise as the factor inducing hearing loss in musicians (16, 17). Pharmaceutical interventions for the treatment of musicians’ hearing loss have been proposed by Wartinger et al. (18). Behar et al. reported on studies using pure tone audiometry but focused on the technique of noise measurement (19). They provided recommendations about noise measurement but did not draw conclusions about the prevalence of musicians’ hearing loss. All of the mentioned studies are not systematic reviews. The only systematic review on the topic is by Di Stadio et al., published in 2018 (20). This thorough review involved a subgroup analysis of pop/rock and classical music musicians. The review included 41 studies and pooled the results from a sample of 4,618 professional musicians. The authors reported a prevalence of musicians’ hearing loss of 63.5% for pop/rock musicians and 32.8% for classical music performers in the frequency range of 3,000–6,000 Hz. A limitation of the review was the heterogeneity of the retrieved studies. The pooling of the results includes a high risk of bias since studies with low quality have a high impact. Furthermore, the proportion of affected individuals relative to the original sample size (prevalence) can be obscured by pooling the results, as well as by grouping the results into different subgroups containing different instrumentalists. With a new methodical approach that preserves the original prevalence findings, we sought to update the research question.

Furthermore, it is important to consider the criteria that distinguish a well-conducted study and the publication of meaningful data. This starts with the design of the study (e.g., cohort size) and extends to the execution of audiometry (e.g., environment, noise breaks, and use of a standardized audiometer), evaluation of the raw data (e.g., age correction or at least its reporting), and preparation of the data for publication (e.g., reporting hearing loss in dB and by frequency).

We did not aim to compare occupational risk assessments; rather, our goal was to determine the prevalence of music-induced hearing loss and its characteristics among musicians.

## Methods

We conducted a literature search following the Preferred Reporting Items for Systematic reviews and Meta-Analyses (PRISMA) guidelines (21), using the search terms *(hearing loss OR hearing impairment OR hearing difficulties OR acoustic trauma) AND (musician).* This search was performed between 14 August 2023 and 2 January 2025, for a final update. We searched the databases such as MEDLINE, Embase, Cochrane Library, and Google Scholar. For the latter, we restricted the search results to a total of 300 entries. The abstracts and titles from the Google Scholar result list were excluded from HTML files using Wolfram Mathematica 13.1. URLs were used when digital object identifiers (DOIs) could not be retrieved. Each Google Scholar entry was added to the screening list. We also included theses and comparable university works to reduce the risk of bias. We applied no filters and included all years of publication. The review was not registered, and the protocol was not published in advance.

The inclusion criteria were as follows: an examination of musicians (mostly professional), and majority age of participants (population), exposure to music performance (exposure), and original research that included the use of pure tone audiometry (outcome).

The exclusion criteria included singers as the study population, music exposure in a leisure context, and publication types such as case report studies, systematic reviews, reports, grey literature, and languages other than English or German. Computational translations for other languages yielded inappropriate results. The study design was not a selection criterion as long as audiometry data were reported for musicians. A control group was not required since we focused on the prevalence of noise-induced hearing loss.

**Figure 1:**
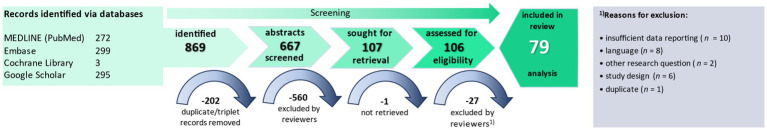
Flowchart. Review process according to the PRISMA guidelines. There were no additional records.

During the review process, we never used automation tools or citation tracking tools. The screening was performed independently by the two authors as reviewers, using xlsx-files. First, the abstracts were screened. The unclear and deviating titles were discussed. In the second step, the full texts were screened in the same manner. The reference lists were not searched systematically, and we did not add any manual entries. The flowchart is presented in Figure 1.

Data extraction was conducted simultaneously and under the constant observation of the other reviewer. The data extraction was based on the following categories: musical genre, study population, number of participants (including the female-to-male ratio) age range of study population, minimum years of musical practice, inclusion and exclusion criteria of the study, instruments used (pure tone audiometry), and results. The results were further specified by the findings (normal hearing, notch, and hearing loss), the affected side of the ear, affected participants or subgroups, and the proportion of the affected participants to the study population. Finally, we assessed the data reporting to determine whether it was detailed and sufficient or poorly done with regard to the specified criteria, including reporting of frequency range, hearing loss in dB, and age correction. In addition, the study design (cross-sectional vs. longitudinal), the kind of data acquisition, and the reporting of raw data were also evaluated. Assessing the risk of bias was not necessary because of the descriptive epidemiological approach. We did not aim to conduct statistical subgroup analyses *a priori* but focused on the results of the musicians’ pure tone audiometry, as described previously using the PEO criteria. As there are no formal quality assessment tools for pure tone audiometry, we decided to limit the results to a descriptive evaluation based on the recommendations of the American Speech–Language–Hearing Association (ASHA). Except for the criterion “age correction,” which was added *post hoc*, all extracted categories were defined primarily.

Studies that included only healthy participants for another primary research issue (e.g., tinnitus) were not included in the final analysis since the prevalence of hearing loss may be biased. Furthermore, the findings that were solely based on comparisons (subgroup, ear side) were excluded since they did not contain prevalence data for the study population.

To conduct a systematic analysis, we grouped the findings into four musical genres: classical music, rock/pop/jazz, traditional music, and military and marching band music. The unreported studies were assigned to the group “unknown.”

For statistics and graphs, we used Wolfram Mathematica 13.1. Descriptive statistics consisted of stacked histograms showing the distribution of quantitative data (number of participants and years of practice). For the distribution of sex, we calculated the percentage values and plotted them in a sorted bar chart. The pure tone audiometry results were categorized into four groups as follows: (1) values with frequency and hearing loss reporting, (2) values with frequency but without hearing loss in dB reporting, (3) values without frequency but with dB hearing loss reporting, and (4) values with neither frequency nor hearing loss reporting. All plot data were categorized by genre, and the resulting counts were collected. A color scale was used for visual coding of the count numbers. These values were plotted in a “conventional” audiogram layout. This approach ensured that no data had to be transformed and that partially missing data could also be reported.

For the visualization of the proportion of the affected persons, we calculated the percentage values and plotted sorted bar charts with opacity decreasing in relation to the maximum study population. Inferential statistics were employed where necessary, using Wolfram Mathematica 13.1. To compare the distribution of categorical variables with more than 50 values (e.g., sex distribution), we used Pearson’s chi-squared test, with *α* = 0.05.

## Results

### Selection of the sources of evidence

Reviewer 1 included 89 studies after screening the titles and abstracts and discussing all unclear cases during the retrieval process (*n* = 72, 14% of all screened studies), while reviewer 2 included 109 studies. The intersection rate for the 89 retrievals was 100%, whereas the final inclusion rate was 89/107 and 109/107 after clarifying the 20 remaining titles. One title could not be retrieved in full text; therefore, 106 titles were assessed in full text. After discussing 19 unclear retrievals, both reviewers included the same 79 studies (see Figure 1).

Some studies did not report pure-tone audiometry results in the context of noise-induced hearing loss because of other research questions (22–27). One case report was excluded because it lacked representativeness (28). One study included a mixed occupational cohort and did not report results specific to the musicians’ subgroup (29). Another study only included healthy participants (30). One study (31) reported the same data as another study already included in this review (32).

### Characteristics of the sources of evidence

**Figure 2:**
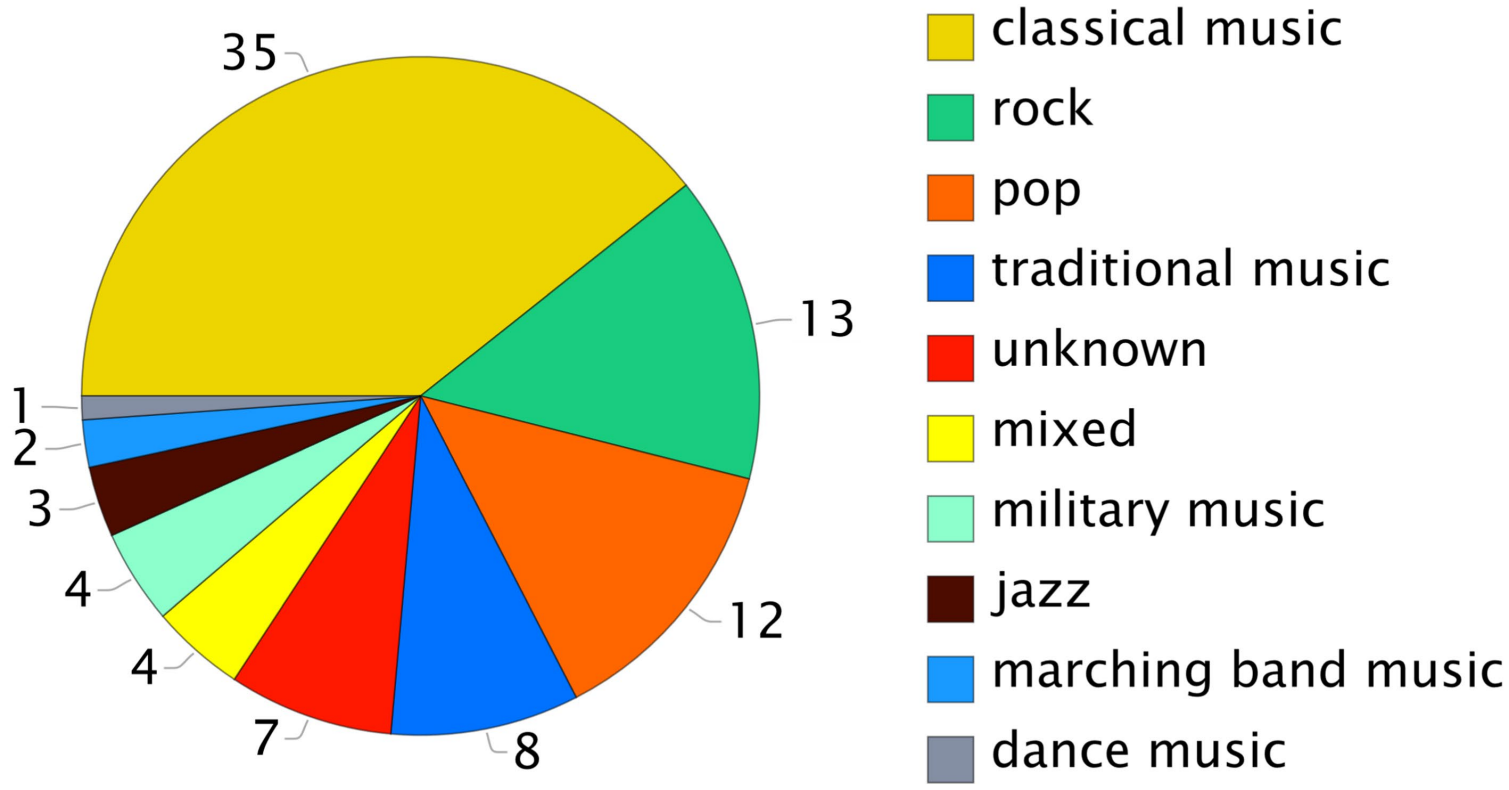
Pie chart showing the distribution of the identified studies by genre.

**Figure 3:**
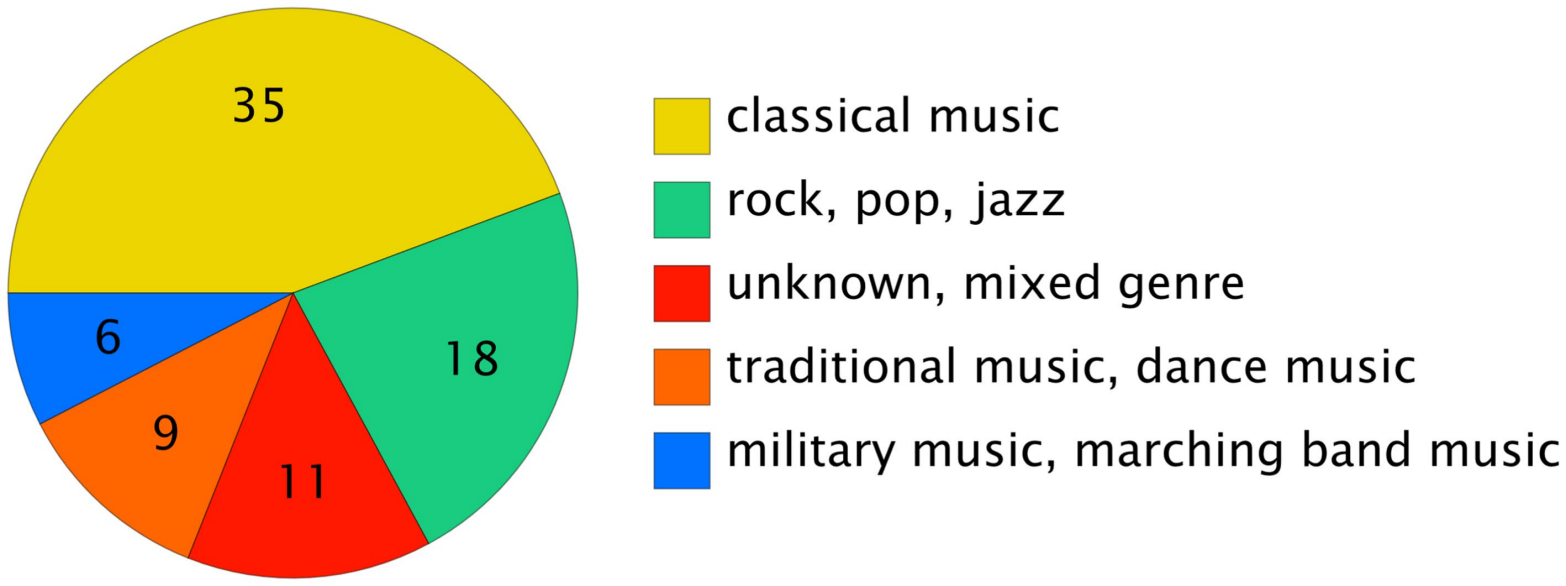
Pie chart of grouping. The retrieved studies were categorized into five genre groups.

The majority of the studies assessed classical music, rock, pop and jazz. Some studies focused on traditional instruments and military or marching band music (see Figure 2). Four studies assessed different genre groups, whereas six studies did not report the genre. We regrouped the genres into five main categories (see Figure 3).

**Figure 4:**
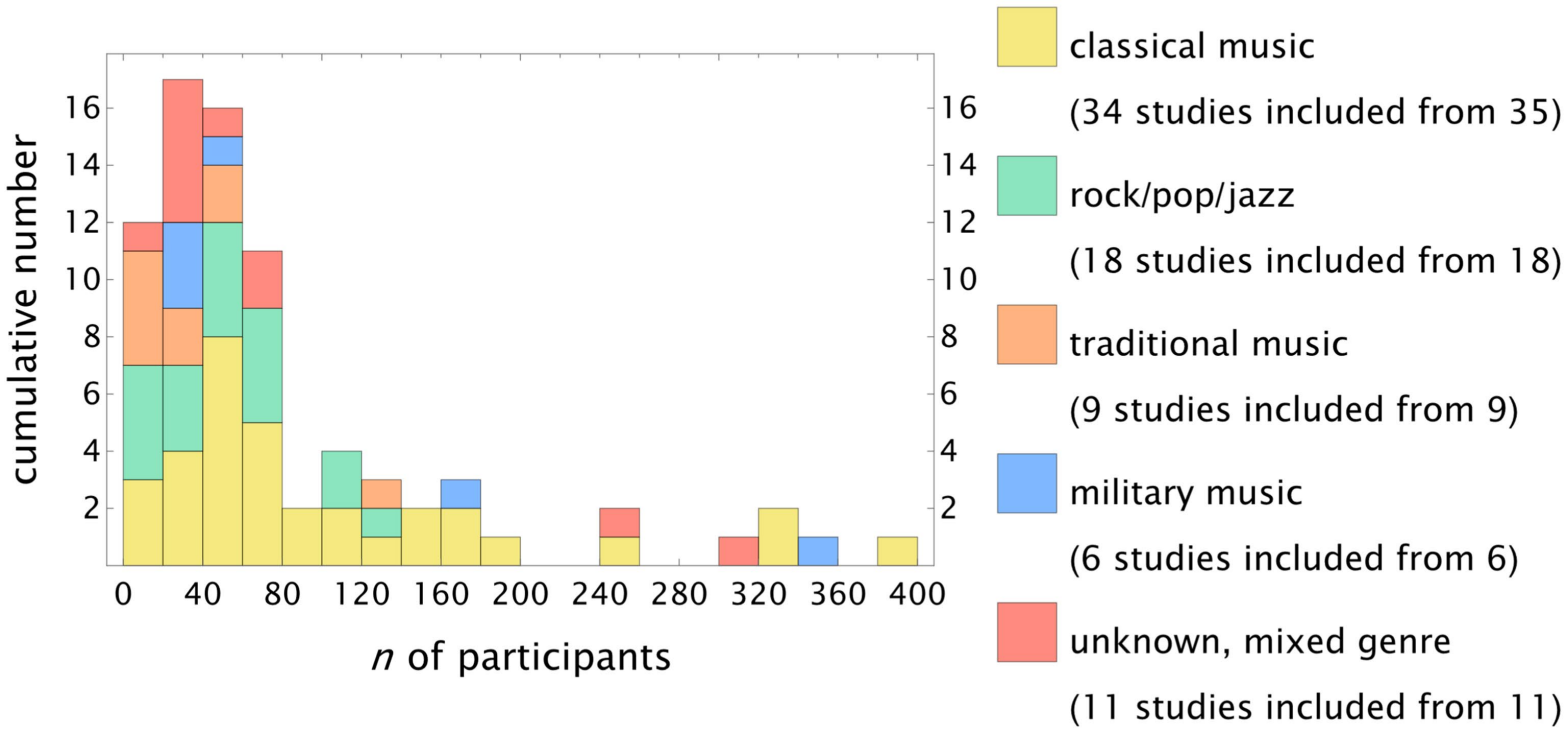
Stacked histogram showing the number of participants. The median was 56 (IQR 31–111).

The overall median number of participants was 52 (IQR 30-109). The distribution is shown in Figure 4. We selected a stacked histogram to visualize the overall distribution and the subgroup results. Compared to the overall distribution, there were more studies in the classical genre in the fourth quartile, with 11 studies having >111 participants (32.4% of *n*_classical music_).

**Figure 5:**
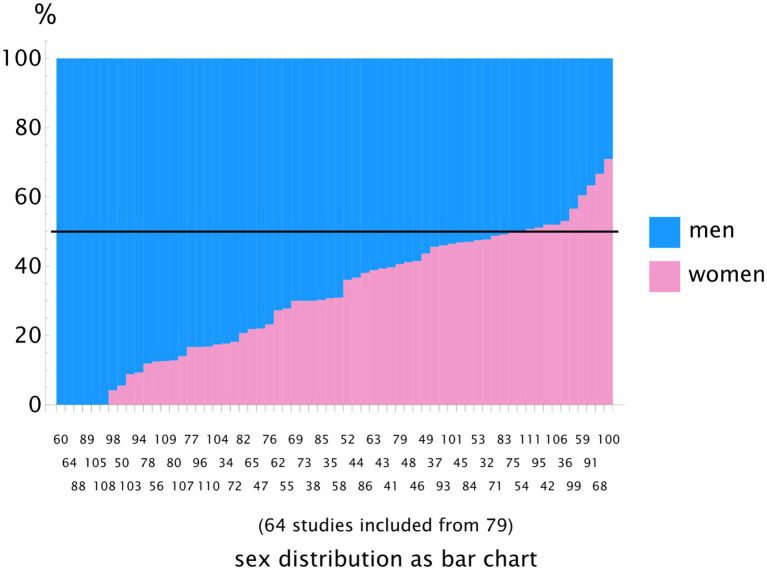
Bar chart showing the sex distribution across all studies. Only a few studies included more female participants than male participants. The majority of the studies had a significantly higher proportion of male participants. The genre subgroup analysis revealed the same distribution, making this overall distribution representative. The numbers on the bars correspond to the literature reference list.

The sex distribution showed a significant majority of male participants, with a median value of 69% (IQR 53–83%) (see Figure 5). The analysis yielded χ^2^(9) = 36.3, *p* < 0.001. The median female proportion was 31% (IQR 17–47%). Figure 5 shows a sorted bar chart displaying the data from all retrieved studies reporting the sex distribution. The 50% value is marked by a bold line as a visual guide. The subgroup analysis showed a similar distribution and is included in the online supplementary material in our repository (33).

**Figure 6:**
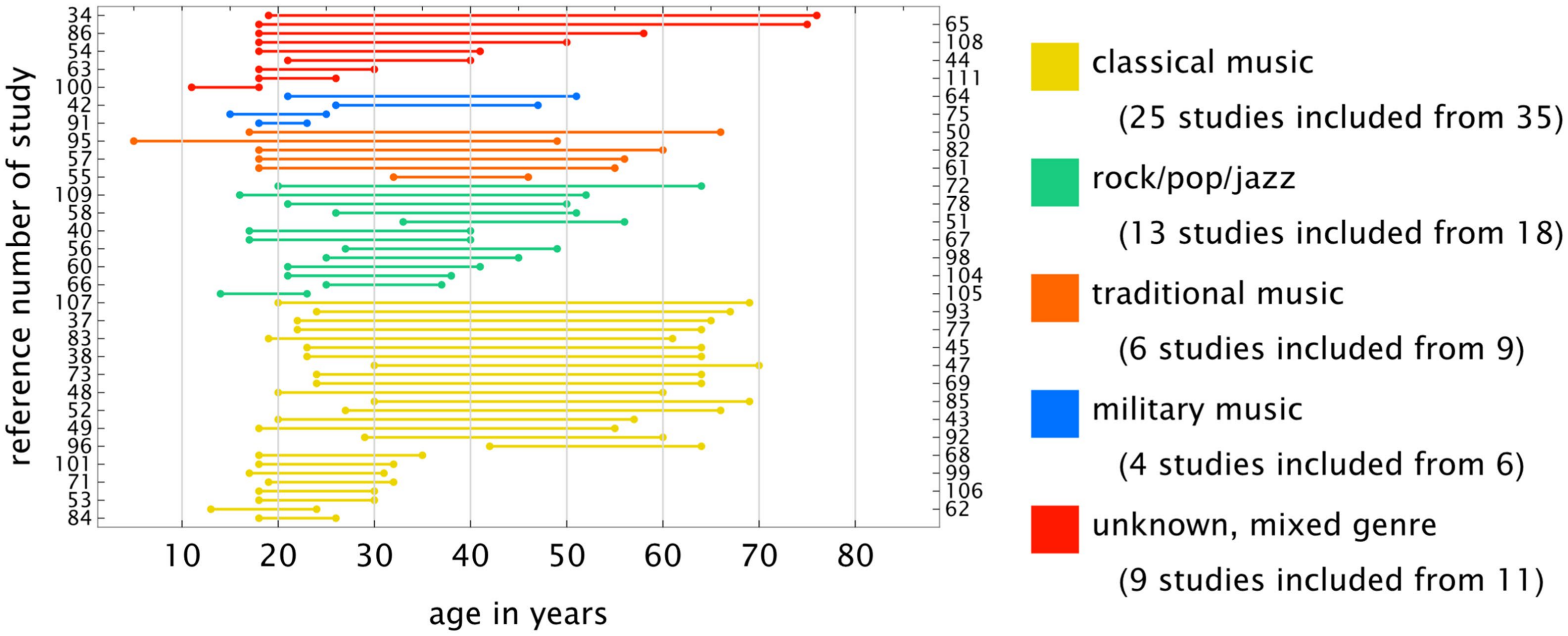
Age range distribution. Many studies focused exclusively on students. Genre-specific studies including individuals over 70 years were lacking. In particular, the studies on non-classical music genres lacked a representative age range. The numbers on the lines correspond to the literature reference list.

The age range differed significantly. Figure 6 plots the number lines for the retrieved age ranges grouped by genre. As can be seen, many studies focused on young participants (students). Studies on rock, pop, and jazz genres did not include participants over 50 years of age, and studies on classical music did not include those over 60–70 years. None of the studies investigated retired musicians.

**Figure 7:**
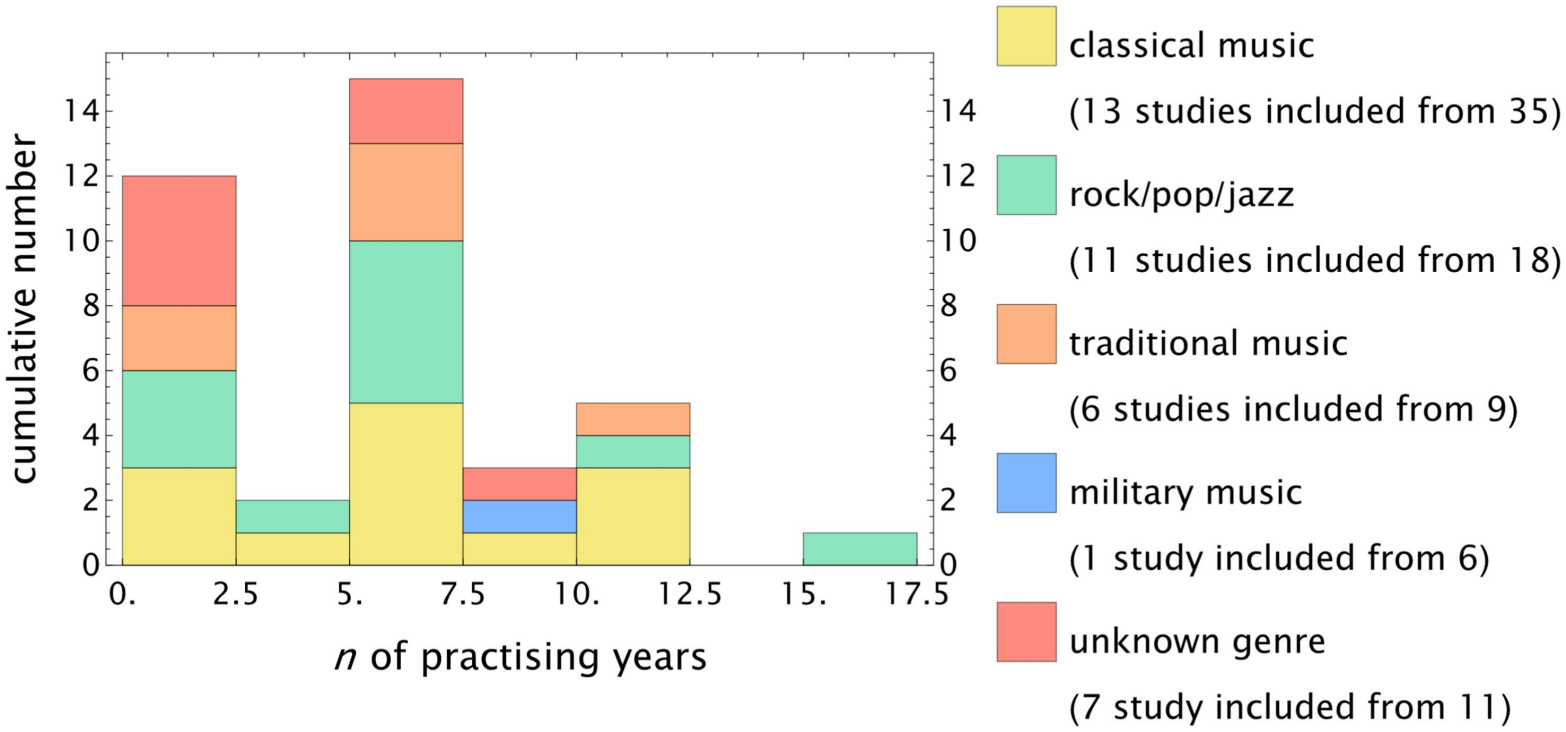
Stacked histogram of minimum years as a practicing musician. The majority of the studies reported a minimum of 5 years of musical practice. Only a few studies investigated longer minimum exposure times.

Some studies reported the number of years spent as practicing musicians is minimal, as shown in Figure 7, which presents a stacked histogram of the subgroup results. The majority of these studies included participants with more than 5 years of musical practice. As mentioned, some studies focused on students with less exposure to music. Only six studies included study participants with more than 10 years of musical practice.

A total of 17 studies reported that they performed age correction for audiometry data (34–50), while 2 reported that they did not (51, 52). Additionally, 60 studies did not specify whether they corrected for age (32, 53–111).

### Results of the sources of evidence

**Figure 8:**
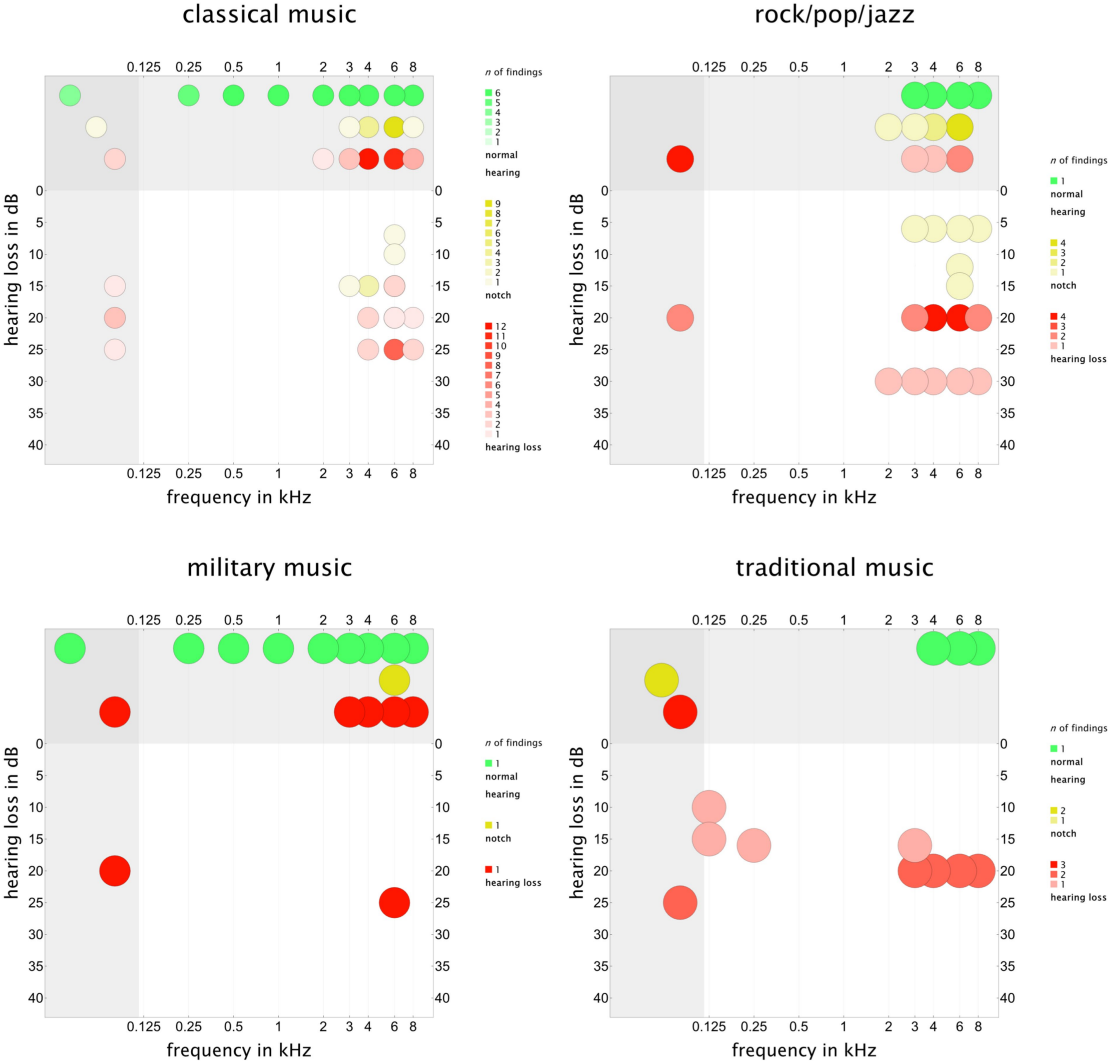
Frequency distribution of the retrieved hearing examination results in the audiogram according to the genre subgroups. Values in the left grey box were published without frequency reporting. Values in the upper grey box lacked dB reporting. Values in the overlapping region, the dark grey box at the top left corner, were reported without frequency and hearing loss in dB. The color scale indicates the number of results found for the corresponding point value. For this purpose, we distinguished between the findings with normal hearing (green), notch (yellow), and hearing loss (red) in the audiogram. Some studies had numerous findings, each counted to detect a common cluster. The corresponding summary table is available online in the repository. This figure should not be interpreted as a prevalence cluster. To analyze the prevalence of music-induced hearing loss, refer to Figures 9–13.

For further analysis, we extracted key results from the retrieved studies, as described in Methods. The summary table is published online in the repository (33). Initially, we aimed to determine whether the results of the studies, which could be numerous, formed clusters based on frequency and threshold shift. As shown in the summary table in the repository^1^, the reporting of hearing loss in relation to frequency range and hearing loss in dB was not consistent. We counted each report in a two-dimensional array for the two parameters and included missing values as well. Afterward, we plotted the results in a bubble plot, with a color scale indicating the frequency of the retrieved findings for each genre (see Figure 8). A total of 10 studies found normal hearing in audiometry of the participants in the classical music group (37, 43, 48, 53, 71, 73, 79, 84, 99, 106). In addition, 10 studies reported a notch configuration in the frequency range of 4,000–6,000 Hz (36, 38, 41, 45, 46, 62, 70, 71, 76, 93, 101), and up to 12 results from 7 studies showed a hearing loss >15–20 dB in the same frequency range (39, 44, 52, 83, 92, 96, 107). The intersection range of the results without dB hearing loss reporting (32, 35, 38, 39, 47, 85, 90, 101, 107) and those without frequency reporting (49, 99) lay exactly in the same area. This cluster suggested an increased risk for musicians in the classical genre to develop hearing loss in the predisposed frequency range.

Concerning the musicians in the rock, pop, and jazz genres, only one study found normal hearing for all participants (51). The majority of the studies showed hearing loss or notch configurations in the frequency range of 3,000–8,000 Hz (56, 66, 74, 78, 94, 101, 104). The frequency range was broader, and hearing loss >20 dB was more frequent in musicians of these genres than in the classical genre group, as commonly expected.

The data availability for the genres of military music and marching band music was limited. A cluster of hearing loss was observed in the frequency range of 4,000–6,000 Hz (41, 75, 89, 91, 103, 107). The same was true for the traditional genre (44, 50, 55, 57, 59, 61, 82, 88, 108, 110), as shown in Figure 8.

### Descriptive synthesis of the results

**Figure 9:**
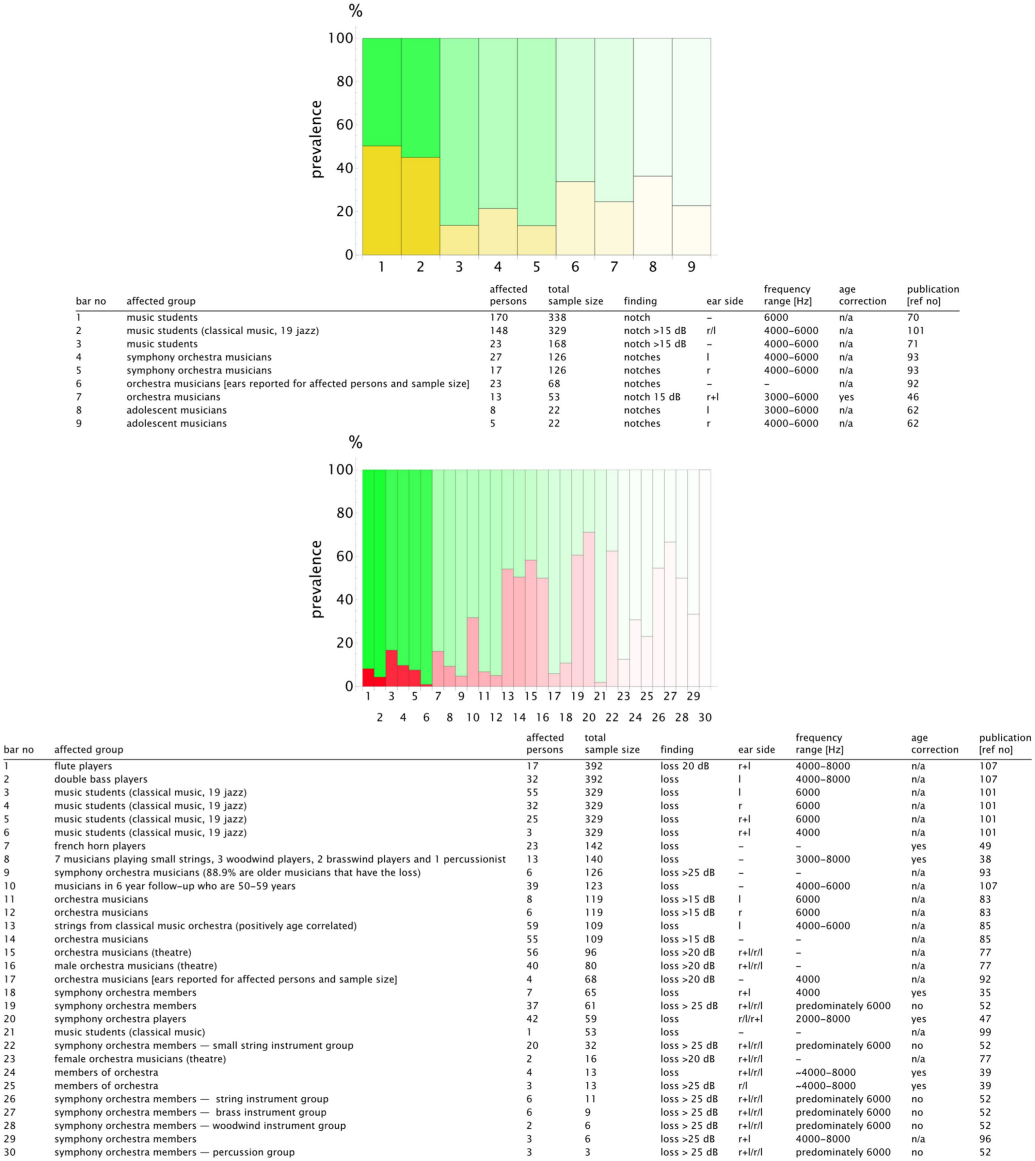
Bar chart showing the number of individuals with notch configurations (yellow) and hearing loss (red) in relation to the sample size for the **classical music group**. The opacity decreases in relation to the study with the maximum sample size (*n*_study_/*n*_max_), meaning that the bars with higher transparency represent the studies with lower validity. The bar number corresponds to the table row below the chart. In this way, the finding can be attributed to the corresponding publication. A detailed result list with DOI links is available in the repository (33).

**Figure 10:**
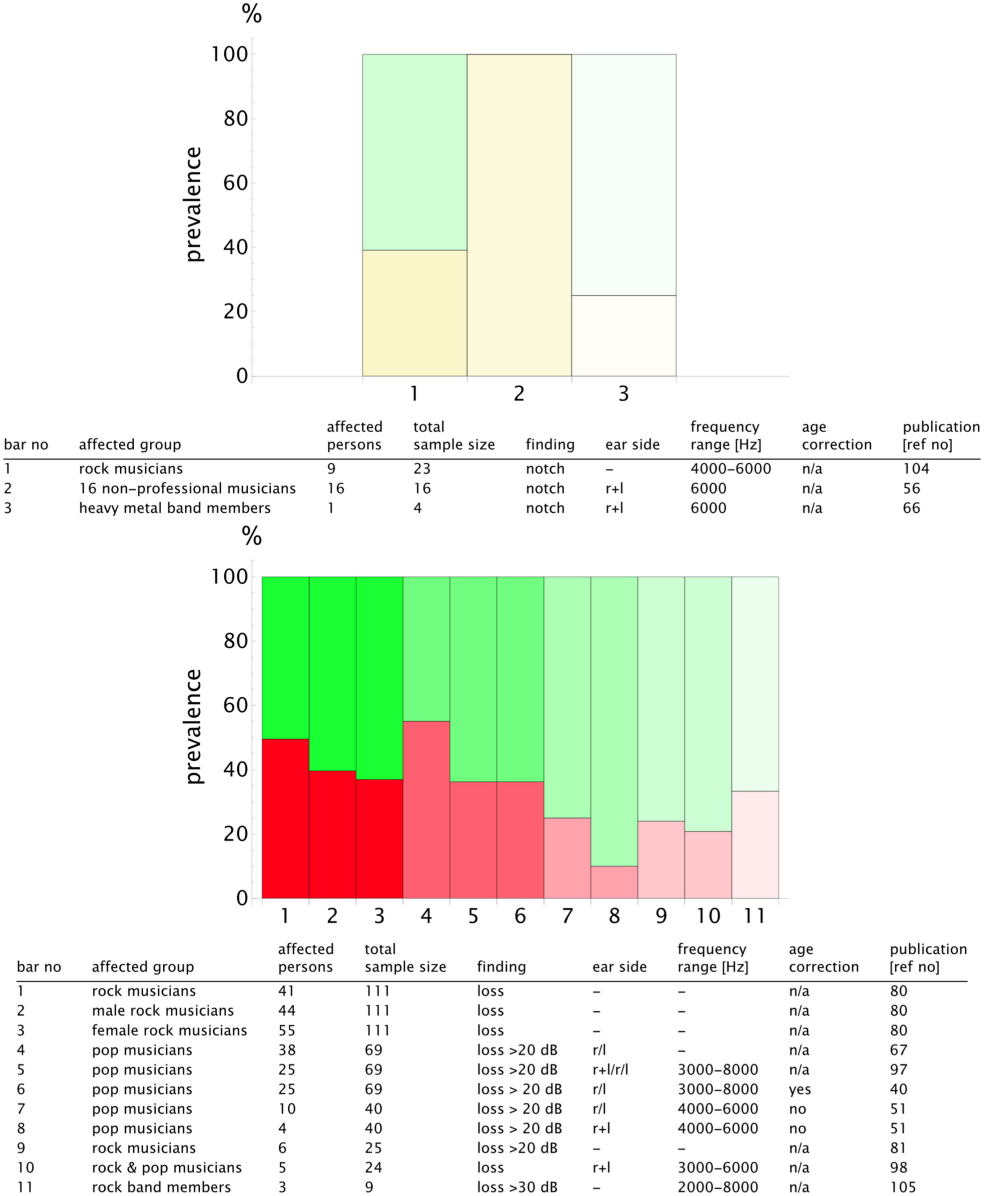
Bar chart showing the number of individuals with notch configurations (yellow) and hearing loss (red) in relation to the sample size for the genre **rock/pop/jazz**. The opacity decreases in relation to the study with the maximum sample size (*n*_study_/*n*_max_), meaning that the bars with higher transparency represent the studies with lower validity. The bar number corresponds to the table row below the chart. In this way, the finding can be attributed to the corresponding publication. A detailed result list with DOI links is available in the repository (33).

**Figure 11:**
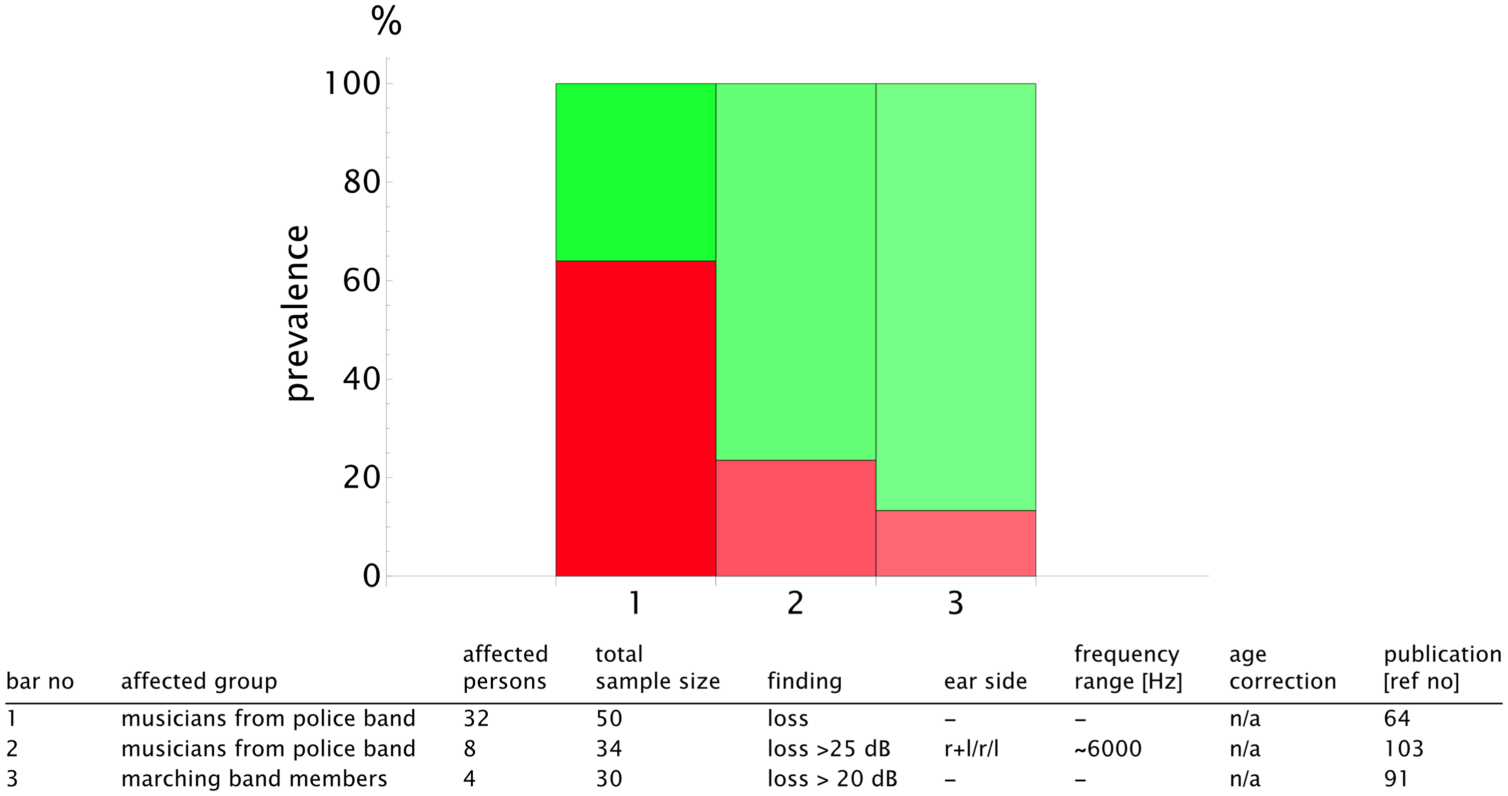
Bar chart showing the number of individuals with hearing loss (red) in relation to the sample size for the genre **military music**. The opacity decreases in relation to the study with the maximum sample size (*n*_study_/*n*_max_), meaning that the bars with higher transparency represent the studies with lower validity. The bar number corresponds to the table row below the chart. In this way, the finding can be attributed to the corresponding publication. A detailed result list with DOI links is available in the repository (33).

**Figure 12:**
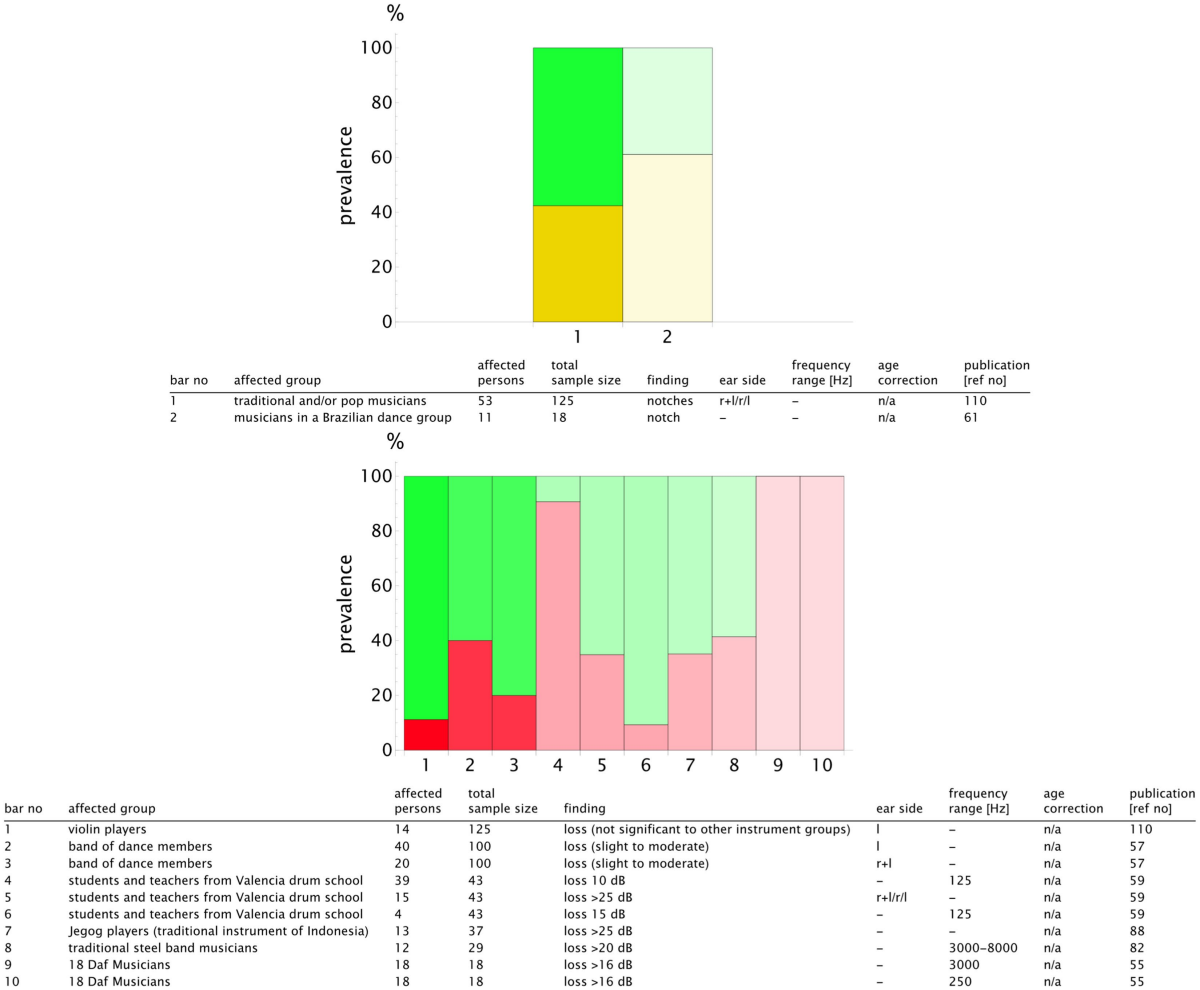
Bar chart showing the number of individuals with notch (yellow) and hearing loss (red) in relation to the sample size for the genre **traditional music**. The opacity decreases in relation to the study with the maximum sample size (*n*_study_/*n*_max_), meaning that the bars with higher transparency represent the studies with lower validity. The bar number corresponds to the table row below the chart. In this way, the finding can be attributed to the corresponding publication. A detailed result list with DOI links is available in the repository (33).

**Figure 13:**
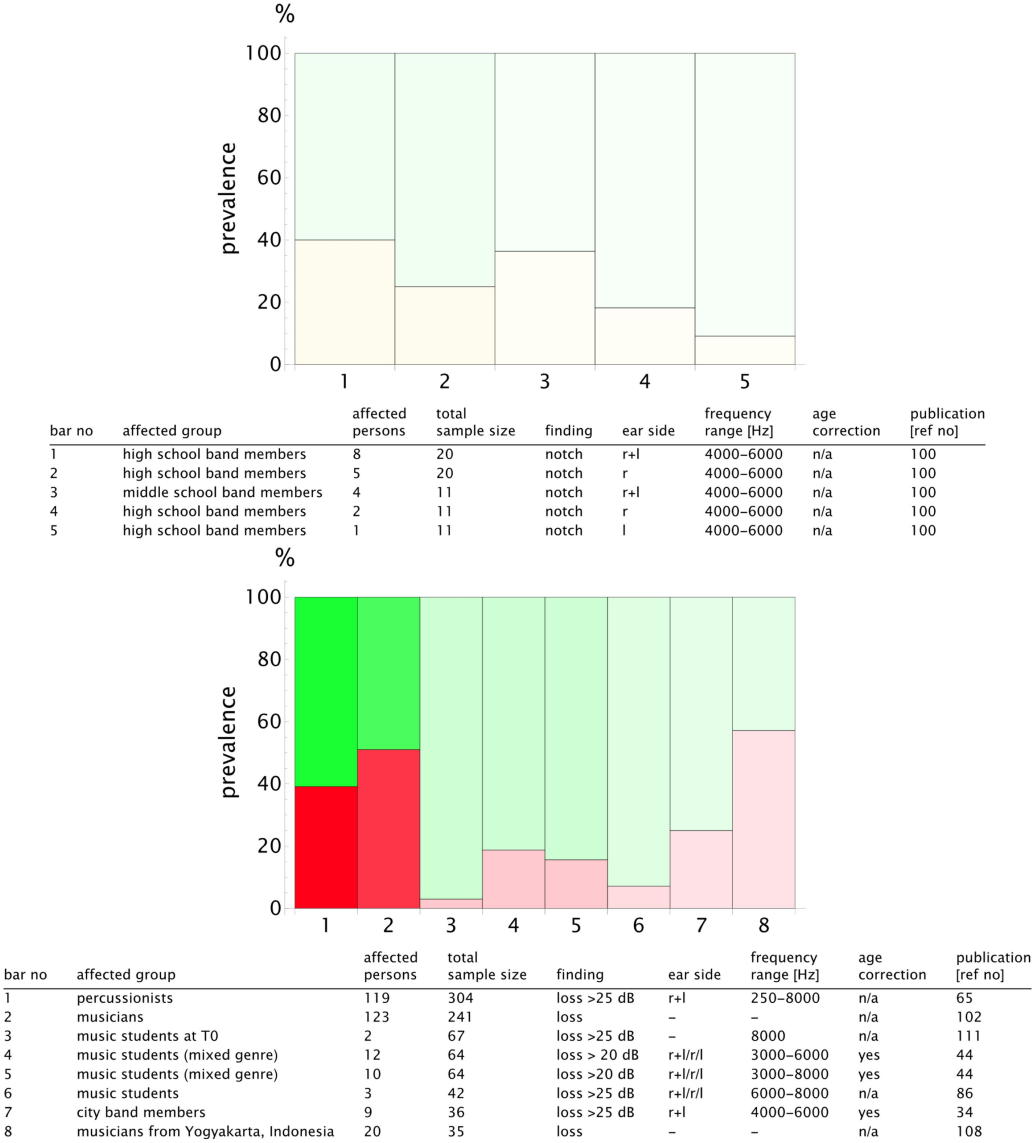
Bar chart showing the number of individuals with notch (yellow) and hearing loss (red) in relation to the sample size for the **unknown and mixed genre** group. The opacity decreases in relation to the study with the maximum sample size (*n*_study_/*n*_max_), meaning that the bars with higher transparency represent the studies with lower validity. The bar number corresponds to the table row below the chart. In this way, the finding can be attributed to the corresponding publication. A detailed result list with DOI links is available in the repository (33).

For a more detailed analysis, we published the complete list of the study results and the summary table in our repository with valid DOIs for each study (33). An extract is presented in Figure 9 for the classical music genre. We plotted the prevalence values of the studies reporting notch configurations and those reporting hearing loss. To highlight the sample size, we used transparency that increased with the decreasing number of the participants. Notch configurations were observed in 20–50% of the musicians, whereas hearing loss prevalence was more scattered, ranging from 5 to 70% (see Figure 9). The rock, pop, and jazz musicians showed notch configurations in 20–100% of the cases, with hearing loss within the range of 20–60% (Figure 10). The number of retrieved studies for the military music and traditional music genre groups was low. The prevalence of hearing loss ranged from 10 to 60% for the military music group and from 10 to 100% for the traditional music group (Figures 11, 12). Some studies did not report the genre or had mixed genres without subgroup reporting. These are listed separately (Figure 13). A clear allocation of hearing loss to one ear could not be systematically detected in all subgroup analyses, as reported in the bar chart tables.

## Discussion

The peer review process with two blinded reviewers yielded 79 studies, according to the PRISMA guidelines. The studies were heterogeneous in terms of the genre, publication of the results, and audiogram data corrected for age. The majority of the retrieved studies focused on young, male musicians with little exposure time. Older or retired musicians, as well as women, were underrepresented or missing. The summary table can be found as an online supplementary material in our repository (33).

For the classical genre, some studies found no hearing loss in their study population, while others found notch configurations or hearing loss >15 dB in the frequency range of 4,000–6,000 Hz. This indicates a higher risk of hearing loss for classical musicians. The prevalence of notch configurations and hearing loss varied between 5 and 70%. A total of 20–60% of rock, pop, and jazz musicians tended to have a higher risk of hearing loss >20 dB in the range of 3,000–8,000 Hz, as expected. The data for military music and traditional music were limited, although an increased risk of hearing loss was also found in the 4,000–6,000 Hz range.

These findings are consistent with an analysis of diagnoses from 2,227 musicians based on data from 7 million German health-insured individuals, which found a hazard ratio of 3.51 (95% Confidence Interval: 2.82 to 4.21) for noise-induced hearing loss in musicians compared to non-insured musicians (5). Concerning the prevalence of noise-induced hearing loss in musicians, a report published in 2009 showed that 58% of musicians in the classical genre and up to 49% in the rock/pop genre were affected (112). The systematic review by Di Stadio et al., presented in the Introduction section, reported hearing loss in 63.5% of rock/pop musicians and 32.8% of classical music performers. Instead of focusing on a mean value, we adopted a holistic approach for reporting the range of the prevalence findings. The findings from the other studies are consistent with the range of our results, as shown in Figure 9 through Figure 12.

Notch configurations and hearing loss in the high-frequency range of 4,000–6,000 Hz are characteristic of noise-induced hearing loss (113–115). Speech-frequency hearing impairment in the range of 500–4,000 Hz is much more common in the general population, with a prevalence of 14.1% in the USA (116). This study also assessed the prevalence of bilateral hearing impairment in relation to occupational noise exposure, defined as exposure “at work to loud sounds or noise for four or more hours, several days a week”. The findings were as follows: no exposure: 14% (95% CI: 13–16), exposure to noise “so loud that they had to raise their voice to be heard” for more than 5 years: 28% (95% CI: 21–37), and exposure to noise “so loud that they had to shout to be heard” for more than 5 years: 43% (95% CI: 35–51). Compared to the retrieved studies, which mostly focused on exposure for more than 5 years, musicians may have an increased prevalence of hearing loss, especially in the rock, pop, and jazz genres (20–60% vs. 14.1%). This prevalence is within the range observed in occupationally exposed individuals: 28 and 43%. However, in our results, the prevalence of hearing loss among musicians in the classical music genre was too broad to draw meaningful conclusions (5–70%). All the studies identified in this review were original works investigating musicians. Only a few had sample sizes >300 and a cohort study design, such as the study by Karlson et al. (107). As shown in Figure 9 through Figure 12, the reporting of hearing loss prevalence depends on different factors. These include the composition of the affected group, which may consist of different instrumentalists; the reporting of audiometry results, which may include dB hearing loss and frequency range; and the type of age correction applied. To discuss these factors, we need to take a closer look at some publications from the classical genre that provide detailed information about them.

Obeling and Poulsen found no signs of hearing loss in 57 musicians from four Danish orchestras (37). They corrected the audiograms using ISO 1999, taking into account the number of years of musical practice, playing hours per week, and the average sound level. The authors published averaged audiogram plots with standard deviations. They reported results for ear-side and instrument groups but did not include a comparison by sex. The authors emphasized that the data were not representative since the sample size was small. Assuming 90 musicians in a symphony orchestra, the participation rate in this study was 15%.

Toppila et al. assessed 63 musicians from 4 symphony orchestras, resulting in a participation rate of approximately 17% (41). The authors corrected for age, noise exposure, and sex using ISO 1999-1990. The audiometry results for both ears were plotted with 95% confidence intervals. Box and whisker charts reported the corresponding z-scores for comparison between a noise-exposed and a non-exposed standard population. They did not find any differences in the musician group compared to the non-exposed population. The authors compared the mean differences in hearing loss between two subgroups: musicians with high exposure (L_ex_ > 100) and musicians with low exposure (L_ex_ < 100). The hearing loss values were significantly higher in the range of 1,000–6,000 Hz for the high-exposure subgroup.

Wegner et al. assessed 40 classical music instrumentalists and reported a participation rate of 30% (48). They reported averaged hearing levels with standard deviations for both ears for the instrumental groups, as well as the use of hearing protection devices. The values were age-corrected based on a preceding study from 1967. Sex comparison was not reported. The authors found normal hearing in the sample.

Eaton and Gillis found notch configurations in the high-frequency range in 13 of 53 musicians from a Canadian symphony orchestra (46). The participation rate was approximately 50%. The audiogram data were corrected using ISO 1999-1990 (as described previously). The averaged audiometry results were compared for sex and instrument groups. Differences in the ear side could not be found.

Kähäri et al. had a sample size of 140 participants from two Swedish orchestras, with a participation rate of 79% (38). They reported mean values with standard deviations, as well as median and range for both ears. The plots show the 10th and 90th percentiles, as well as groupings by musical instrument, sex, and age. The data were not corrected for age, but the age groups are reported in detail. The findings indicated no severe hearing loss among musicians but highlighted a more frequent notch configuration in the men’s audiogram and slightly worse hearing in the percussion and woodwind players.

Participation rates are often low, which may introduce a bias, with individuals experiencing hearing loss less likely to participate in the study. Reporting averaged hearing loss values carries the risk of underestimating subgroup differences and may result in the loss of information about the distribution of the original audiogram data. Age correction often relies on ISO 1999-1990 references (37, 41, 46), and noise exposure must be calculated beforehand (37). The heterogeneity of assessments demands standardized methods and prospective study designs. Longitudinal studies are completely lacking, making it impossible to assess the lifetime prevalence of hearing loss among musicians. Prospective multicenter studies are needed to address this research question.

Knowledge about music-induced hearing loss is crucial since hearing ability is essential for a musician’s work. Occupational hearing loss often results from continuous exposure and may go unnoticed by the affected person (12, 117). This highlights the importance of regular audiometric examinations for musicians, in accordance with the laws of the respective country. Only through this approach can music-induced hearing loss be detected and preventive measures be implemented. For the latter, systematic and scientific evaluation is needed to avoid accidental harm. Organizational measures should also be considered since they are easy to implement. For example, reducing the playing volume during rehearsals could help mitigate risk. To avoid temporary threshold shifts from prolonged exposure, loud passages could be played at the end of a rehearsal. After rehearsal, sufficient recuperation time should be ensured. Music presents a complex challenge that rarely allows for the use of conventional preventive hearing protection measures typical in occupational health.

## Data Availability

Publicly available datasets were analyzed in this study. This data can be found here: Firle C, Richter AH. Repository for supplementary data to musicians’ hearing loss—a systematic review 2024 (https://doi.org/10.5281/zenodo.11225123).

## Footnotes

^1^ https://github.com/Carl-Firle/Review_Musicians-_Hearing_Loss/blob/main/Summary%20Table.md
